# Associations of neighborhood deprivation with breast cancer tumor genomics, targeted treatment use, and survival

**DOI:** 10.1007/s10549-026-08068-3

**Published:** 2026-09-01

**Authors:** Emily L. Podany, Lorenzo Foffano, Lorenzo Gerratana, Arielle J. Medford, Natalie K. Heater, Eleonora Nicolò, Shaili Tapiavala, Letizia Pontolillo, Annika Putur, Diana A. Jaber, Katherine Clifton, Nitin Katakam, Sarah Addison, Marla Lipsyc-Sharf, Carolina Reduzzi, Foluso O. Ademuyiwa, Fabio Puglisi, William J. Gradishar, Cynthia X. Ma, Aditya Bardia, Massimo Cristofanilli, Andrew A. Davis

**Affiliations:** 1 Department of Medicine, Washington University in St. Louis, 660 South Euclid Ave., Campus Box 8056, St. Louis, MO 63110 USA; 2 Department of Medical Oncology, CRO Aviano, National Cancer Institute, IRCCS, Aviano, Italy; 3 Department of Medicine, University of Udine, Udine, Italy; 4 Department of Medicine, Massachusetts General Hospital Cancer Center, Boston, MA USA; 5 Department of Medicine, Northwestern University, Chicago, IL USA; 6 Department of Medicine, Weill Cornell Medicine, New York, NY USA; 7 Department of Medicine, UCLA Health, Los Angeles, CA USA; 8 Medical Oncology, Department of Translational Medicine and Surgery, Universitá Cattolica del Sacro Cuore, Rome, Italy

**Keywords:** Disparities, Breast cancer, Neighborhood deprivation, ctDNA

## Abstract

**Purpose:**

Neighborhood environments appear to influence breast cancer biology and outcomes. This study evaluated somatic, treatment, and outcome differences by Area Deprivation Index in patients with metastatic breast cancer.

**Methods:**

Retrospective, population-based cohort study using clinical and genomic data gathered between 2015 and 2024 at four academic institutions in the United States. The outcomes were differences in circulating tumor DNA mutation profiles, PI3K inhibitor use, and survival between patients with metastatic breast cancer living in high deprivation (Area Deprivation Index ≥ 60 by national rank) and low deprivation (< 60) neighborhoods.

**Results:**

Among 1127 patients with metastatic breast cancer, 335 (29.7%) lived in high deprivation areas. These patients were more likely to have *TP53* mutations (Odds ratio 1.49, 95% Confidence Interval 1.07–2.08, *P* = 0.018). Among hormone receptor-positive, HER2-negative patients eligible for PI3K inhibitors, those from high deprivation areas were less likely to receive them (17.4% vs. 36.7%, *p* = 0.02). Median survival from the time of circulating tumor DNA testing was significantly shorter in the high deprivation group (24 months versus 28 months, *p* = 0.04) and for Black patients in the high deprivation group versus Black patients in low deprivation group and all White patients (15 months versus 25–28 months, *p* = 0.02).

**Conclusions:**

We found that patients with metastatic breast cancer living in high deprivation neighborhoods were more likely to have *TP53* mutations, an indicator of aggressive disease biology, less likely to receive PI3K inhibitors, and had shorter overall survival compared to patients living in low deprivation neighborhoods by Area Deprivation Index.

**Supplementary Information:**

The online version contains supplementary material available at https://doi.org/10.1007/s10549-026-08068-3.

## Introduction

In the United States (US) and worldwide, there are significant racial disparities in breast cancer diagnosis, treatment, and outcomes [1–10]. These disparities are multifactorial and include socioeconomic disadvantages. Black communities in the US experience a higher level of poverty, which affects healthcare access, physical and psychological wellbeing, education, cancer-specific outcomes, and exposure to environmental pollution [11–21]. 

In our prior work, we demonstrated differences in circulating tumor DNA (ctDNA) somatic mutation profiles between Black and White patients with metastatic breast cancer (MBC) that did not fully account for disparities in outcomes; however, this research did not include lived environment, including poverty, social determinants of health, and neighborhood characteristics [22]. Neighborhood deprivation has been shown to have a potential impact on breast cancer, including tissue inflammation, tissue DNA methylation, gene expression, hormone receptor status, and incidence and mortality [23–33]. The Neighborhood Atlas Area Deprivation Index (ADI) is a validated measure to explore socioeconomic disadvantage at a neighborhood level in the US [34]. ADI includes 17 measures such as poverty, employment, and education. It stratifies these into a state ranking from 1 to 10 and a national ranking from 1 to 100 using nine-digit zip codes, with higher levels representing greater socioeconomic deprivation. We hypothesized that ctDNA somatic mutation profiles and survival outcomes may differ between patients with MBC living in high and low ADI neighborhoods.

As oncologists increasingly utilize precision oncology in treating patients with MBC, more patients will receive targeted treatments such as phosphoinositide 3-kinase inhibitors (PI3Ki). PI3Ki are Food and Drug Administration (FDA) approved for patients with hormone-receptor positive (HR+), HER2 negative (HER2-) MBC harboring eligible *PIK3CA*,* AKT*, and *PTEN* mutations [35–37]. *PIK3CA* is a particularly common mutation present in up to 40% of HR*+* HER2- tumors. There are limited data on racial and socioeconomic disparities in the use of targeted treatments in patients with MBC [22]. We focused this study on the effects of ADI, which we hypothesized may impact PI3Ki access and use.

To explore these hypotheses, we performed a large multi-institution retrospective cohort study to determine whether patients with MBC have differences in somatic profiles, PI3Ki use, and outcomes in low versus high deprivation neighborhoods by ADI.

## Materials and methods

### Design

This retrospective cohort study was designed and reported using the Strengthening the Reporting of Observational Studies in Epidemiology (STROBE) guidelines and was conducted with approval from the institutional review boards at Washington University in St. Louis, Weill Cornell Medicine, Northwestern University, and Massachusetts General Hospital with a waiver of informed consent. The study was conducted in accordance with the Health Insurance Portability and Accountability Act (HIPAA) and all clinical and genomic information were deidentified and stored in a HIPAA-compliant manner under data use agreements.

### Participants

All patients with diagnosed MBC and at least one ctDNA result treated between 2015 and 2024 were identified at Washington University in St. Louis, Massachusetts General Hospital, Weill Cornell Medicine, and Northwestern University, collaborating through the nonprofit group Precision Medicine Action for Cancer. To reduce variation between tests, patients were chosen who received ctDNA testing using the Guardant360 assay (Guardant Health). Clinical information was gathered through electronic medical record (EMR) review including self-reported race and ethnicity, 9-digit zip code, age, breast cancer subtype, treatment information, sites of metastases on imaging at the time of baseline ctDNA testing, and survival after first ctDNA test. Patients were excluded if they had no zip code data, had a PO Box listed instead of an address, or were living outside of the catchment area states for each cancer center, as this may indicate they had a separate oncologist closer to home.

### ctDNA testing and pathway analysis

The Guardant360 assay detects pathogenic mutations, variants of uncertain significance, and synonymous alterations, and the sensitivity and detection thresholds have been previously described [38, 39]. The assay detects deletions, insertions, and single nucleotide variants (SNVs) in up to 83 genes, copy number variants in 19 genes, and fusions in 11 genes. We used the definitions of oncogenic pathways from The Cancer Genome Atlas and limited our pathway analysis to only genes detectable by the Guardant360 assay.

### Area deprivation index

We extracted the 9-digit zip code for each patient’s home address, converted it into state and national rankings using the 2022 ADI state zip code linkages, and merged the ADI scores with the clinical and genomic data. We chose to utilize national rankings rather than within-state rankings because there is inter-state variability in average wealth. We divided the cohort into patients living in high deprivation (HDep, ADI ≥ 60 by national rank) and low deprivation neighborhoods (LDep, ADI < 60 by national rank) based on prior research [40–44]. 

### PI3Ki use and survival

We determined receipt of PI3Ki by EMR review of patients with HR+, HER2- MBC and eligible mutations. We did not include hemoglobin A1c in our assessment. Patients were evaluated by ADI and use of PI3Ki in the second line or beyond, either through clinical trial enrollment or after the Food and Drug Administration approval of the first PI3Ki, alpelisib, in May 2019 [35]. We calculated survival from the first ctDNA test to last known EMR contact with the patient or death with a data cutoff of 12/31/2024.

### Statistical analysis

Clinical and pathologic variables were reported using descriptive analyses. Categorical variables were reported as frequency distributions, whereas continuous variables were described through median and interquartile ranges (IQRs). Clinical and genomic differences between HDep and LDep were assessed using univariate and multivariate logistic regression to estimate odds ratios (ORs) and 95% confidence intervals (CIs). Only gene alterations with at least five pathogenic events were considered for inclusion. Baseline ctDNA was not universally collected at the time of metastatic diagnosis, and thus we have adjusted for line of treatment at the time of baseline ctDNA testing. Multivariate analysis was adjusted for age, race, subtype, site of metastatic disease, line of treatment at the time of baseline ctDNA testing, *de novo* disease status, and mean allelic frequency. As these were exploratory analyses, we did not apply a correction for multiple testing. Differences in the use of PI3Ki in the second line of treatment and beyond between HDep and LDep were assessed using the chi-square test. Overall survival (OS) was defined from the time of baseline ctDNA collection to death from any cause, with data censored at last follow-up if the patient was still alive. Differences in OS were assessed using the log-rank test and Cox proportional hazards regression models and displayed using Kaplan-Meier plots. Only pathogenic alterations based on OncoKB were included in the logistic and Cox proportional hazards regression models. Statistical analysis was performed from November 2024 to January 2025 using Stata, version 16.1 (StataCorp LLC), JMP, version 16 (SAS Institute Inc), and R, version 4.1.0 (R foundation for Statistical Computing).

## Results

### Cohort characteristics

**Fig. 1:**
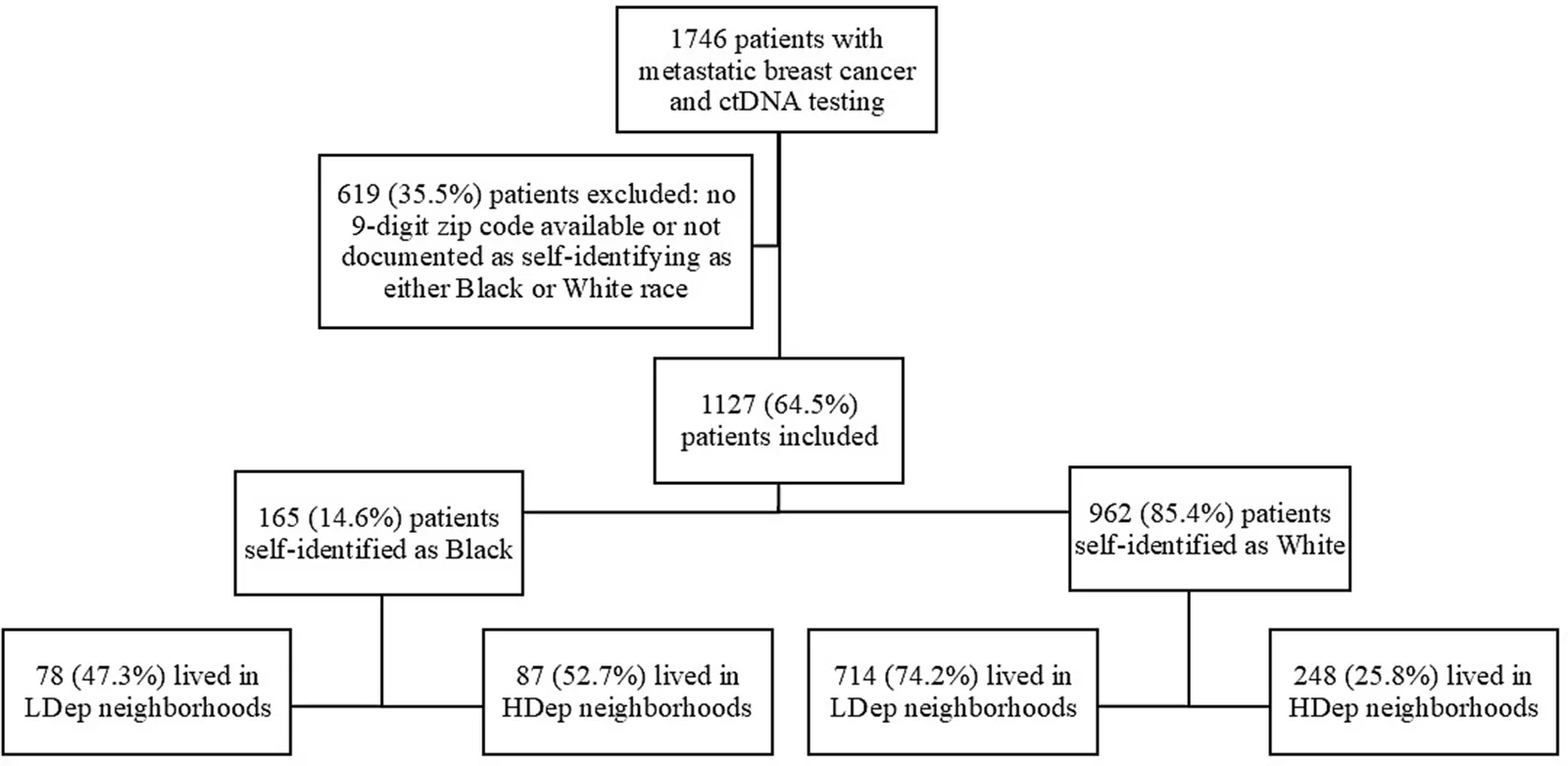
CONSORT diagram depicting the creation of the cohorts

This retrospective cohort included 1127 patients with MBC, at least one ctDNA result, and a 9-digit zip code available (Fig. 1). The patients were treated at Washington University (*N* = 634), Massachusetts General Hospital (*N* = 313), Weill Cornell Medicine (*N* = 109), and Northwestern University (*N* = 71). The mean ADI across the cohort was 40.5 (standard deviation 28.7). 335 patients (29.7%) lived in HDep neighborhoods (Table 1). All patients were female. 165 patients (14.6%) self-identified as Black, 962 patients (85.4%) self-identified as White (Table 1). Due to the small number in each category, patients self-identified as American Indian, Asian, Pacific Islander or with unknown identified race were excluded from the analysis.

**Table 1:** Characteristics of the cohort in the general population and in the two subgroups (LDep and HDep), with corresponding Fisher’s exact test analysis. Missing data were not reported

	*N* (%)	LDep (792, 70.28%)	HDep (335, 29.72%)	Fisher test
**Race**				
White	962 (85.36%)	714 (90.15%)	248 (74.03%)	< 0.001
Black	165 (14.64%)	78 (9.85%)	87 (25.97%)	
**Subtype**				
HR+/HER2-	737 (75.20%)	530 (74.44%)	207 (77.24%)	0.59
HER2+	98 (10.00%)	75 (10.53%)	23 (8.58%)	
TNBC	145 (14.80%)	107 (15.03%)	38 (14.18%)	
**Histotype**				
Ductal	729 (81.63%)	520 (80.00%)	209 (86.01%)	< 0.001
Lobular	125 (14.00%)	91 (14.00%)	34 (13.99%)	
Mixed	39 (4.37%)	39 (6.00%)	0 (0.00%)	
**Visceral**				
No	523 (48.92%)	324 (44.02%)	199 (59.76%)	< 0.001
Yes	546 (51.08%)	412 (55.98%)	134 (40.24%)	
**Bone**				
No	384 (34.13%)	257 (32.53%)	127 (37.91%)	0.08
Yes	741 (65.87%)	533 (67.47%)	208 (62.09%)	
**Nodes**				
No	750 (66.67%)	498 (63.04%)	252 (75.22%)	< 0.001
Yes	375 (33.33%)	292 (36.96%)	83 (24.78%)	
**Skin/Soft tissues**				
No	962 (85.51%)	641 (81.14%)	321 (95.82%)	< 0.001
Yes	163 (14.49%)	149 (18.86%)	14 (4.18%)	
**De Novo**				
No	734 (74.14%)	522 (73.83%)	212 (74.91%)	0.73
Yes	256 (25.86%)	185 (26.17%)	71 (25.09%)	
**Therapy line**				
1	355 (37.25%)	248 (36.52%)	107 (39.05%)	0.51
2	203 (21.30%)	151 (22.24%)	52 (18.98%)	
>=3	395 (41.45%)	280 (41.24%)	115 (41.97%)	

Abbreviations: LDep, low deprivation neighborhoods; HDep, high deprivation neighborhoods; ER, estrogen receptor; PR, progesterone receptor; HR, hormone receptor; HER2+, HER2 postiive

Black patients were significantly more likely to be from HDep areas than White patients (OR 3.21, 95% CI 2.29–4.50, *p* < 0.001) (Supplementary Table 1). In LDep neighborhoods, White patients were more likely to have bone metastases than Black patients (69.2% vs. 51.3%, *p* = 0.001) and Black patients were more likely to have *de novo* MBC (37.1% vs. 25.1%, *p* = 0.04) (Table 2). In HDep neighborhoods, Black patients were more likely to have skin or soft tissue metastases than White patients (Table 3). Other characteristics including subtype and line of were similar between races (Tables 2 and 3).

**Table 2:** Characteristics of the LDep cohort overall and in the two racial subgroups (Black and White), with corresponding Fisher’s exact test analysis. Missing data were not reported

	*N* (%)	White (714, 90.15%)	Black (78, 9.85%)	Fisher test
**Subtype**				
HR+/HER2-	530 (74.44%)	487 (75.39%)	43 (65.15%)	0.08
HER2+	75 (10.53%)	63 (9.75%)	12 (18.18%)	
TNBC	107 (15.03%)	96 (14.86%)	11 (16.67%)	
**Histotype**				
Ductal	520 (80.00%)	469 (79.36%)	51 (86.44%)	0.42
Lobular	91 (14.00%)	85 (14.38%)	6 (10.17%)	
Mixed	39 (6.00%)	37 (6.26%)	2 (3.39%)	
**Visceral**				
No	324 (44.02%)	296 (44.25%)	28 (41.79%)	0.70
Yes	412 (55.98%)	373 (55.75%)	39 (58.21%)	
**Bone**				
No	257 (32.53%)	219 (30.76%)	38 (48.72%)	0.001
Yes	533 (67.47%)	493 (69.24%)	40 (51.28%)	
**Nodes**				
No	498 (63.04%)	445 (62.50%)	53 (67.95%)	0.34
Yes	292 (36.96%)	267 (37.50%)	25 (32.05%)	
**Skin/Soft tissues**				
No	641 (81.14%)	580 (81.46%)	61 (78.21%)	0.48
Yes	149 (18.86%)	132 (18.54%)	17 (21.79%)	
**De Novo**				
No	522 (73.83%)	483 (74.88%)	39 (62.90%)	0.040
Yes	185 (26.17%)	162 (25.12%)	23 (37.10%)	
**Therapy line**				
1	248 (36.52%)	229 (36.82%)	19 (33.33%)	0.84
2	151 (22.24%)	137 (22.03%)	14 (24.56%)	
>=3	280 (41.24%)	256 (41.16%)	24 (42.11%)	

Abbreviations: LDep, low deprivation neighborhoods; HR, hormone receptor; HER2+, HER2 positive

**Table 3:** Characteristics of the HDep cohort overall and in the two racial subgroups (Black and White), with corresponding Fisher’s exact test analysis. Missing data were not reported

	*N* (%)	White (248, 74.03%)	Black (87, 25.97%)	Fisher test
**Subtype**				
HR+/HER2-	207 (77.24%)	153 (77.27%)	54 (77.14%)	0.82
HER2+	23 (8.58%)	18 (9.09%)	5 (7.14%)	
TNBC	38 (14.18%)	27 (13.64%)	11 (15.71%)	
**Histotype**				
Ductal	209 (86.01%)	153 (85.00%)	56 (88.89%)	0.44
Lobular	34 (13.99%)	27 (15.00%)	7 (11.11%)	
**Visceral**				
No	199 (59.76%)	152 (61.29%)	47 (55.29%)	0.33
Yes	134 (40.24%)	96 (38.71%)	38 (44.71%)	
**Bone**				
No	127 (37.91%)	92 (37.10%)	35 (40.23%)	0.60
Yes	208 (62.09%)	156 (62.90%)	52 (59.77%)	
**Nodes**				
No	252 (75.22%)	190 (76.61%)	62 (71.26%)	0.32
Yes	83 (24.78%)	58 (23.39%)	25 (28.74%)	
**Skin/Soft tissues**				
No	321 (97.18%)	241 (97.18%)	80 (91.95%)	0.036
Yes	14 (4.18%)	7 (2.82%)	7 (8.05%)	
**De Novo**				
No	212 (74.91%)	160 (76.19%)	52 (71.23%)	0.40
Yes	71 (25.09%)	50 (23.81%)	21 (28.77%)	
**Therapy line**				
1	107 (39.05%)	79 (38.54%)	28 (40.58%)	0.91
2	52 (18.98%)	40 (19.51%)	12 (17.39%)	
>=3	115 (41.97%)	86 (41.95%)	29 (42.03%)	

Abbreviations: HDep, high deprivation neighborhoods; HR, hormone receptor; HER2+, HER2 positive

30.9% of patients had their baseline ctDNA testing at the time of diagnosis (349/1127), 18.0% (203/1127) were tested at progression after first line treatment, and 53.1% (598/1127) were tested either after second line treatment or beyond. There were no differences in the timing of baseline ctDNA sample collection by ADI status or race.

### Tumor differences and PI3Ki use

**Fig. 2:**
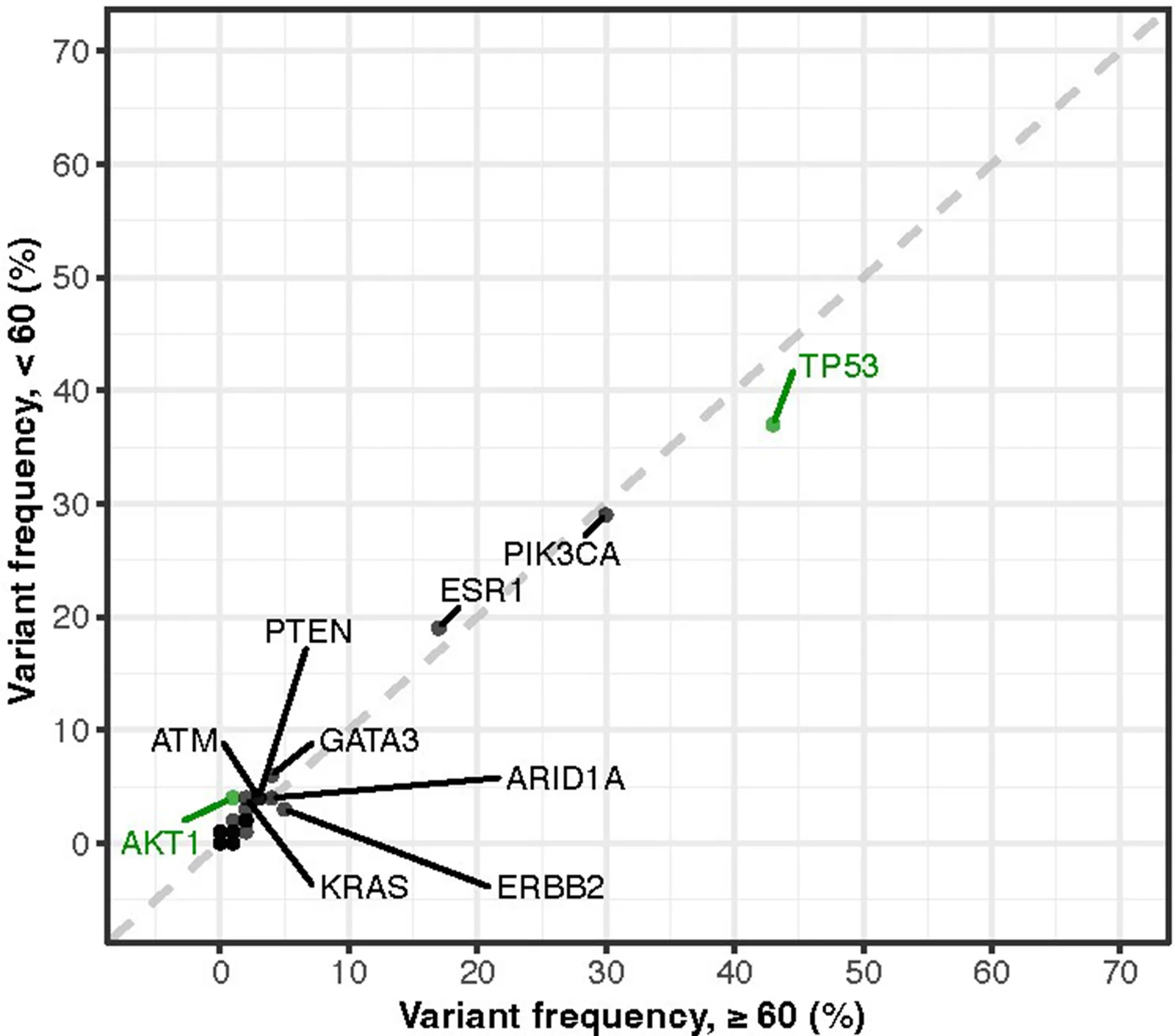
Variant frequencies of the top 10 SNVs in somatic circulating tumor DNA profiles between patients living in high ADI (≥ 60) and low ADI (< 60) neighborhoods. snv = single nucleotide variant, cnv = copy number variant

Patients living in HDep neighborhoods were significantly less likely to present with visceral (OR 0.53, 95% CI 0.41–0.69, *P* < 0.001), node (OR 0.56, 95% CI 0.42–0.75, *P* < 0.001), and soft tissue (OR 0.19, 95% CI 0.11–0.33, *P* < 0.001) metastases. No differences emerged according to line of treatment, *de novo* status and breast cancer subtype (Supplementary Table 1). Patients living in HDep neighborhoods were significantly more likely to have tumor protein p53 (*TP53*) SNVs (OR 1.29, 95% CI 1.00-1.69, *P* = 0.048) and less likely to have AKT serine/threonine kinase 1 (*AKT1*) SNVs (OR 0.37, 95% CI 0.14–0.96, *P* = 0.042) on univariate analysis, with 31 of the 36 patients with *AKT1* SNV residing in LDep (Supplementary Table 2, Fig. 2). These findings were confirmed on multivariate analysis (OR 1.49, 95% CI 1.07–2.08, *P* = 0.018 and OR 0.31, 95% CI 0.11–0.87, *P* = 0.026, respectively) (Supplementary Table 3). Considering oncogenic pathways, patients in the HDep group remained significantly associated with *TP53* SNVs in both univariate (OR 1.34, 95% CI 1.03–1.73, *P* = 0.027) and multivariate analyses (OR 1.67, 95% CI 1.20–2.33, *P* = 0.002) (Supplementary Tables 4–5). There was no difference in *PIK3CA* mutation incidence by ADI when controlled for subtype.

A total of 136 patients with HR+ HER2- MBC harbored activating *PIK3CA* mutations and were eligible for PI3Ki therapy, either in the second line and beyond or through clinical trial participation. Forty-six of these patients resided in HDep neighborhoods (33.8%). There was a significantly lower rate of PI3Ki use for patients living in HDep versus LDep neighborhoods (8/46 [17.4%] versus 33/90 [36.7%], Chi-square *p* = 0.02).

### Prognostic and survival disparities

In the LDep group, after variable selection through univariate analysis and adjustment for significant clinical variables (Supplementary Tables 6–7), a significant prognostic impact emerged for *ESR1* SNVs (Hazard ratio [HR] 1.61, 95% CI 1.23–2.11, *P* = 0.<001), *TP53* SNVs (HR 1.67, 95% CI 1.33–2.11, *P* < 0.001), *PIK3CA* CNVs (HR 1.74, 95% CI 1.19–2.55, *P* = 0.005), and *MYC* CNVs (HR 1.91, 95% CI 1.20–2.66, *P* = 0.002) (Supplementary Tables 8–9). When evaluating oncogenic pathways, after selection through univariate analysis (Supplementary Table 10), a significant impact in OS was identified for *P53* SNVs (HR 1.94, 95% CI 1.57–2.41, *P* < 0.001), *PI3K* CNVs (HR 2.14, 95% CI 1.54–2.97, *P* < 0.001), and *MYC* CNVs (HR 2.44, 95% CI 1.74–3.43, *P* < 0.001) (Supplementary Tables 11–12).

Among patients in the HDep group, patients who self-identified as Black had worse prognosis (HR 1.47, 95% CI 1.03–2.10, *p* = 0.034) (Supplementary Table 13). After selection of variables in the univariate analysis (Supplementary Tables 13–14), a significant impact in OS emerged for *PIK3CA* SNVs (HR 1.65, 95% CI 1.20–2.28, *P* = 0.002), *TP53* SNVs (HR 1.79, 95% CI 1.30–2.46, *P* < 0.001), *AKT1* SNVs (HR 6.27, 95% CI 2.27–17.33, *P* < 0.001), *BRAF* CNVs (HR 5.36, 95% CI 2.91–9.87, P = < 0.001), *FGFR1* CNVs (HR 2.34, 95% CI 1.40–3.91, *P* = 0.001) and *MYC* CNVs (HR 4.53, 95% CI 2.64–7.76, *P* < 0.001) (Supplementary Tables 15–16). When we evaluated oncogenic pathways and prognosis, after selection of variables in the univariate analysis (Supplementary Table 17), patients had poorer prognosis in the HDep group with tumors expressing *PI3K* SNVs (HR 1.84, 95% CI 1.33–2.53, *P* < 0.001), *P53* SNVs (HR 1.73, 95% CI 1.26–2.38, *P* = 0.001), *RTK/RAS* CNVs (HR 2.59, 95% CI 1.82–3.67, *P* < 0.001), and *MYC* CNVs (HR 4.53, 95% CI 2.64–7.76, *P* < 0.001) (Supplementary Tables 18–19).

**Fig. 3:**
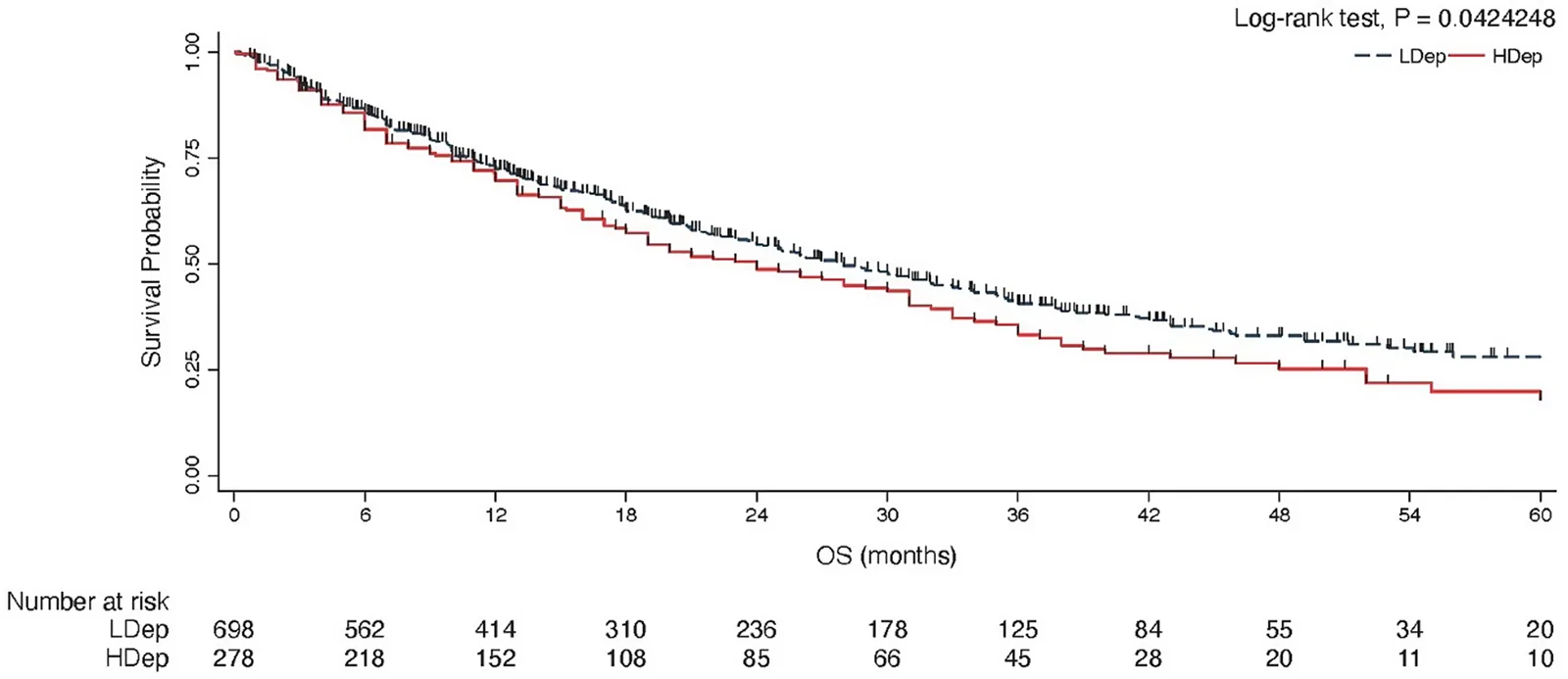
Kaplan-Meier plots for overall survival (OS) by ADI (LDep vs. HDep). Median OS was significantly shorter in the patients living in HDep versus LDep neighborhoods (24 months versus 28 months *p* = 0.04)

**Fig. 4:**
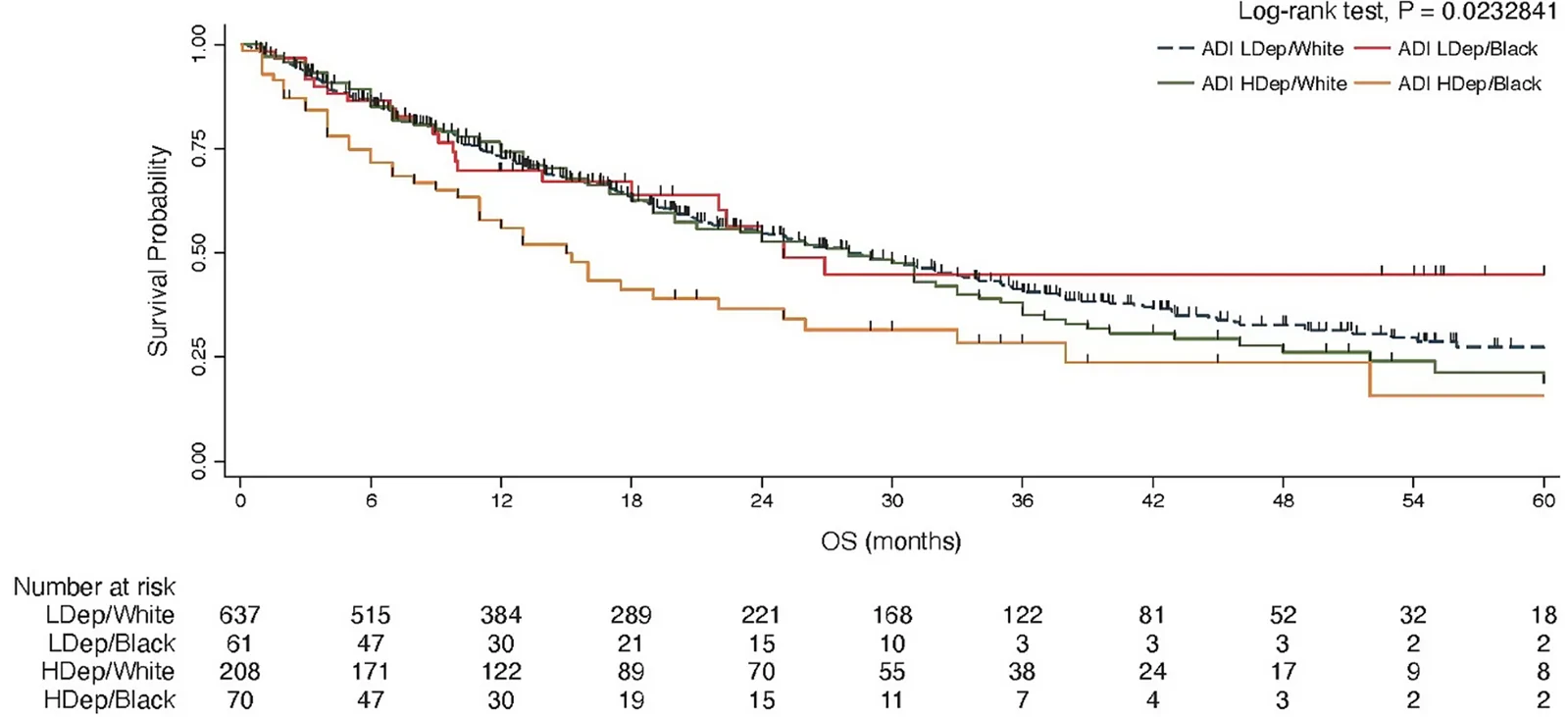
Kaplan-Meier plots for overall survival (OS) by ADI and race. Median OS was 15 months for HDep/Black, 25 months for LDep/Black, 28 months for LDep/White and 28 months for HDep/White (*p* = 0.02)

Median OS was significantly shorter in the patients living in HDep versus LDep neighborhoods (24 months versus 28 months *p* = 0.04). (Fig. 3) Black patients living in HDep neighborhoods had a significantly shorter OS when compared to Black patients living in LDep neighborhoods and White patients living in either HDep or LDep neighborhoods (15 months versus 25 months, 28 months, and 28 months respectively, *p* = 0.02). (Fig. 4)

## Discussion

We describe here our key findings regarding differences in tumor genomic profiles, targeted treatment use, prognosis, and survival by ADI in a large, multi-institution cohort of patients with MBC. Our cohort was racially diverse, multi-state, and included patients living in neighborhoods ranging from 1 to 100 in national ADI ranking. First, we showed a higher rate of somatic *TP53* mutations and lower rates of *AKT1* by ctDNA in patients with MBC in highly deprived neighborhoods versus those in less deprived areas. Our findings support and expand on prior research that used smaller sample sizes and tissue-based assays to show an association between higher frequency of *TP53* mutations in breast cancer tissue and lower socioeconomic status [45, 46]. 

*TP53* is a gene that codes for the tumor suppressor protein p53, which can induce apoptosis and senescence, regulate DNA repair, and trigger cell cycle arrest in damaged cells [47–49]. Mutations in *TP53* can lead to unchecked growth of cancerous or abnormal cells. *TP53* is one of the most common genetic variants in breast cancer tumors, with approximately 30% of cases showing a mutation, and mutations have been shown to be associated with non-Luminal A subtypes, higher stage, higher tumor grade, and worse prognosis [47, 48, 50–54]. Our finding of higher rates of *TP53* in more disadvantaged neighborhoods reveals a potential biological correlate to explain the shorter OS of breast cancer patients living there.

Intriguingly, HDep patients also had lower rates of visceral metastases than LDep patients. This may indicate that the *TP53* mutations are driving more aggressive disease and shorter OS without visceral crises and potentially at an earlier time point. A potentially lower burden of disease but with shorter OS is concerning for potential disparities: treatment choice, rapid progression, performance status, co-morbidities, social determinants of health and consistency of care. Social determinants of health and overall functional status are key to successful treatment, and patients living in HDep neighborhoods have higher rates of food insecurity, sarcopenia, and underlying chronic disease [55–57]. Future studies will be needed to understand the complex biological interplay among lived environment, therapy resistance, somatic profile, co-morbidities, and tumor microenvironment.

*AKT1* encodes a protein that is phosphorylated by PI3K to form AKT/PI3K. This complex is instrumental in multiple cell signaling pathways, including those involved in malignant cell survival. The activation of the AKT pathway can cause resistance to endocrine therapy, and approximately 3% of breast tumors harbor an *AKT1* mutation [58]. *AKT1* mutations are generally enriched in grade 1 breast cancer tumors [59]. When examining HR+ HER2- tumors by intrinsic molecular subtype, luminal A tumors are more likely to have *AKT1* mutations, and these mutations are rare in the more aggressive basal-like subtype [60]. This further suggests that the tumors of the LDep may have an overrepresentation of luminal A versus basal-like subtype. Basal-like subtype breast cancer is more common in Black women, notably has a higher rate of *TP53* mutations, and further investigation into the genomic profiles andoutcomes of specific intrinsic molecular subtypes of breast cancers by ADI is warranted to assess whether patients in HDep neighborhoods may have a higher rate of these more aggressive subtypes despite similar rates of HR+ HER2- and TNBC [61–63]. 

Critically, *AKT1* mutations had a significant impact on prognosis for patients living in HDep neighborhoods, shortening OS. Capivasertib, an oral targeted treatment, inhibits the AKT pathway and is FDA approved for patients with AKT pathway alterations [36]. As patients living in HDep neighborhoods have a lower likelihood of receiving PI3Ki, they may not be receiving the appropriate targeted therapy even when they do have an *AKT1* mutation.

Second, we determined that of patients eligible to receive PI3Ki, those living in higher deprivation areas were less likely to receive PI3Ki compared to lower deprivation areas. Prior research has shown that patients with breast cancer living in disadvantaged neighborhoods have higher rates of omitting radiation after breast conserving surgery [64], longer treatment delays [65], higher rates of early discontinuation of endocrine therapy [66], and receive fewer lines of chemotherapy [67]; however, targeted treatment use disparities in MBC are understudied. We demonstrated that there were disparities in PI3Ki use despite equal incidence of *PIK3CA* activating mutations. This expands on our prior racial disparity work and demonstrates that these inequities are multifactorial and will require a multifaceted approach to address.

Previous research has shown that the rapid development of novel therapies in MBC worsens care disparities, leading to disparities in clinical outcomes [68]. Novel targeted therapies are often expensive, and many precision medicine therapies require testing to determine the presence of a specific genetic mutation or level of protein expression. Patients living in more advantaged neighborhoods are more likely to have access to the newest cancer treatments through geographic proximity and higher socioeconomic status. Cancer centers with clinical trials are located more often in affluent areas, and patients from HDep areas are more likely to travel long distances to receive cancer care [69, 70]. Patients in underserved communities are less likely to enroll in clinical trials for novel treatments and less likely to have private health insurance [71, 72]. The cost of and access to these novel treatments may be influencing patient or provider decision making, and implementation science research designed to address targeted treatment disparities in MBC through outreach and education should be explored in the future for patients living in the most deprived neighborhoods. As barriers to access for PI3Ki were not entirely clear from review of the electronic medical record, we plan to conduct structured patient interviews to elucidate barriers to PI3Ki use in HDep areas as a future direction.

Third, our study showed significantly shorter survival from the time of ctDNA testing for patients living in more highly deprived neighborhoods, especially for Black patients. Studies have shown decreased survival for patients living in disadvantaged neighborhoods with pancreatic [73], lung [74, 75], prostate [76–78], colorectal [79], head and neck [80], central nervous system [81], ovarian [82], and breast cancers [23, 26–29]. Our study adds to these data with a large cohort of diverse patients with MBC who were geographically heterogenous, and separates the survival by race, showing that Black patients from higher deprivation neighborhoods had a significantly shorter OS when compared to White patients living in all neighborhoods or Black patients living in lower deprivation neighborhoods. Black patients living in LDep neighborhoods have the same OS as White patients in either setting, indicating that poverty and neighborhood deprivation modulate race in MBC outcomes. It is not simply a racial disparity; race and neighborhood deprivation appear to interact to produce the shorter OS for Black patients living in HDep. It will be key to examine molecular changes from environmental exposures in laboratory settings alongside public health initiatives in the future.

Our study has several strengths and limitations. The strengths of our work include the size of the cohort, the inclusion of individual-level clinical and genomic data, the use of multiple institutions with catchment areas across several states, the quality of data by using individual chart review for each patient in the EMR, and the breadth of patient ADI across the entire spectrum from first to 100th national percentile. The use of ADI is also a strength as a validated, easily accessible, and widely used measure of neighborhood disadvantage that agrees with other measures of neighborhood deprivation [34, 83]. However, there are inherent limitations to the use of ADI, including over-emphasis on housing prices [84], which is addressed in our cohort by the inclusion of geographically heterogenous patients across multiple states. We were also unable to determine whether patients moved after their interaction with oncology clinics; however, previous research has shown that even highly mobile populations have very similar ADI results between addresses, thus it is acceptable to use the current patient address in the EMR to determine neighborhood disadvantage [85]. Due to the requirement of the presence of 9-digit zip codes for ADI calculation, we were unable to include patients who used PO Boxes, who lived outside of the catchment area and traveled for oncology consultation, or who lacked an address, but this only represented 1% of our total patient population. Another limitation of this study may be reduced generalizability of the findings to the broader U.S. breast cancer population, as all the patients were treated at large academic medical centers. While the sample includes racial and socioeconomic diversity, it may not fully reflect individuals receiving care in other healthcare settings, such as community hospitals or rural clinics. Finally, given that carcinogenesis is a multiyear process, our analysis is limited by using a single time point measure of neighborhood deprivation.

## Conclusion

This multi-institution retrospective cohort study using detailed clinical and genomic data from patients with MBC across multiple states demonstrated differences in tumor genomic profiles indicating aggressive disease biology, PI3Ki use, and survival by ADI. Our findings support the need for further research into the environmental and socioeconomic mediation of breast cancer tumors and prognosis, including laboratory, epidemiological, and implementation science studies.

## Supplementary Information

Below is the link to the electronic supplementary material.

Supplementary Material 1

## Data Availability

The datasets generated and/or analyzed during the current study are not publicly available due to the inclusion of patient genomic data but are available from the corresponding author on reasonable request. The underlying code for this study is not publicly available but may be made available to qualified researchers on reasonable request from the corresponding author.
