# Supplementary material for: Associations of neighborhood deprivation with breast cancer tumor genomics, targeted treatment use, and survival
Source: Breast Cancer Res Treat. 2026 Sep 1;219(2):5. doi: 10.1007/s10549-026-08068-3 (PMC13534212; doi:10.1007/s10549-026-08068-3)
Supplement: Supplementary file 1 [file 10549_2026_8068_MOESM1_ESM.docx]

|  | **OR** | **95% C.I.** | **P>\|z\|** |
| --- | --- | --- | --- |
| **Visceral** | 0.53 | 0.41-0.69 | **<0.001** |
|  |  |  |  |
| **Bone** | 0.79 | 0.61-1.03 | 0.082 |
|  |  |  |  |
| **Node** | 0.56 | 0.42-0.75 | **<0.001** |
|  |  |  |  |
| **Soft tissue** | 0.19 | 0.11-0.33 | **<0.001** |
|  |  |  |  |
| **De novo** | 0.94 | 0.69-1.30 | 0.726 |
|  |  |  |  |
| **Subtype (HR+HER2- reference)** |  |  |  |
| HER2+ | 0.79 | 0.48-1.29 | 0.337 |
| TNBC | 0.91 | 0.61-1.36 | 0.644 |
|  |  |  |  |
| **Therapy line** |  |  |  |
| 2 | 0.80 | 0.54-1.18 | 0.255 |
| >=3 | 0.95 | 0.70-1.30 | 0.758 |
|  |  |  |  |
| **Black** | 3.21 | 2.29-4.50 | **<0.001** |
|  |  |  |  |
| **Age (≥60 vs <60)** | 1.49 | 1.12 – 1.98 | **0.006** |

**Supplementary Table 1.** Univariate analysis of clinical characteristics of the cohorts (LDep vs HDep).

Abbreviations: LDep, low deprivation neighborhoods; HDep, high deprivation neighborhoods; OR, Odds Ratio; C.I, Confidence Interval

|  | **OR** | **95% C.I.** | **P>\|z\|** |
| --- | --- | --- | --- |
| **PIK3CA SNVs** | 1.03 | 0.77-1.36 | 0.851 |
|  |  |  |  |
| **ESR1 SNVs** | 0.89 | 0.64 – 1.25 | 0.520 |
|  |  |  |  |
| **TP53 SNVs** | 1.29 | 1.00-1.69 | **0.048** |
|  |  |  |  |
| **ARID1A SNVs** | 1.05 | 0.56-1.94 | 0.889 |
|  |  |  |  |
| **ERBB2 SNVs** | 1.32 | 0.70-2.46 | 0.384 |
|  |  |  |  |
| **PTEN SNVs** | 0.86 | 0.47-1.74 | 0.680 |
|  |  |  |  |
| **AKT1 SNVs** | 0.37 | 0.14-0.96 | **0.042** |
|  |  |  |  |
| **GATA3 SNVs** | 0.63 | 0.35-1.16 | 0.140 |
|  |  |  |  |
| **BRCA2 SNVs** | 1.09 | 0.55-2.20 | 0.799 |
|  |  |  |  |
| **KRAS SNVs** | 0.53 | 0.24-1.15 | 0.109 |
|  |  |  |  |
| **ATM SNVs** | 0.70 | 0.33-1.49 | 0.358 |
|  |  |  |  |
| **BRAF CNVs** | 1.31 | 0.71-2.40 | 0.379 |
|  |  |  |  |
| **FGFR1 CNVs** | 1.12 | 0.72-1.75 | 0.602 |
|  |  |  |  |
| **CCND1 CNVs** | 0.83 | 0.49-1.41 | 0.501 |
|  |  |  |  |
| **PIK3CA CNVs** | 0.67 | 0.40-1.14 | 0.141 |
|  |  |  |  |
| **EGFR CNVs** | 1.18 | 0.73-1.88 | 0.500 |
|  |  |  |  |
| **MYC CNVs** | 0.78 | 0.45-1.32 | 0.352 |
|  |  |  |  |
| **CCNE1 CNVs** | 1.33 | 0.76-2.34 | 0.316 |

**Supplementary Table 2.** Univariate analysis of single gene alterations in the cohorts (LDep vs. HDep).

Abbreviations: LDep, low deprivation neighborhoods; HDep, high deprivation neighborhoods; SNVs, Single Nucleotide Variants; CNVs, Copy Number Alterations; OR, Odds Ratio; C.I, Confidence Interval

|  | **OR** | **95% C.I.** | **P>\|z\|** |
| --- | --- | --- | --- |
| **Black** | 5.82 | 3.67-9.22 | **<0.001** |
|  |  |  |  |
| **Visceral** | 0.62 | 0.44 – 0.86 | **0.004** |
|  |  |  |  |
| **Node** | 0.82 | 0.58 – 1.15 | 0.254 |
|  |  |  |  |
| **Soft tissue** | 0.14 | 0.07-0.29 | **<0.001** |
|  |  |  |  |
| **Age (≥60 vs <60)** | 1.30 | 0.94 – 1.79 | 0.108 |
|  |  |  |  |
| **Subtype (HR+/HER2- as reference)** |  |  |  |
| **HER2+** | 1.11 | 0.64 – 1.94 | 0.695 |
| **TNBC** | 0.90 | 0.56 – 1.44 | 0.662 |
|  |  |  |  |
| **TP53 SNVs** | 1.49 | 1.07 – 2.08 | **0.018** |
|  |  |  |  |
| **AKT1 SNVs** | 0.31 | 0.11-0.87 | 0.026 |

**Supplementary Table 3.** Multivariate analysis of the cohorts (LDep vs. HDep).

Abbreviations: LDep, low deprivation neighborhoods; HDep, high deprivation neighborhoods; SNVs, Single Nucleotide Variants; CNVs, Copy Number Alterations; OR, Odds Ratio; C.I, Confidence Interval

|  | **OR** | **95% C.I.** | **P>\|z\|** |
| --- | --- | --- | --- |
| **PI3K SNVs** | 0.88 | 0.67-1.16 | 0.376 |
|  |  |  |  |
| **ER SNVs** | 0.88 | 0.64-1.20 | 0.405 |
|  |  |  |  |
| **TP53 SNVs** | 1.34 | 1.03-1.73 | **0.027** |
|  |  |  |  |
| **RTK SNVs** | 0.79 | 0.54-1.15 | 0.213 |
|  |  |  |  |
| **Cell Cycle SNVs** | 0.89 | 0.44-1.81 | 0.754 |
|  |  |  |  |
| **RTK/RAS CNVs** | 1.04 | 0.76-1.41 | 0.816 |
|  |  |  |  |
| **Cell Cycle CNVs** | 0.85 | 0.58-1.24 | 0.394 |
|  |  |  |  |
| **PI3K CNVs** | 0.67 | 0.40-1.14 | 0.141 |
|  |  |  |  |
| **MYC CNVs** | 0.78 | 0.45-1.32 | 0.352 |

**Supplementary Table 4.** Univariate analysis of pathway alterations in the general population (LDep vs. HDep).

Abbreviations: LDep, low deprivation neighborhoods; HDep, high deprivation neighborhoods; SNVs, Single Nucleotide Variants; CNVs, Copy Number Alterations; OR, Odds Ratio; C.I, Confidence Interval

|  | **OR** | **95% C.I.** | **P>\|z\|** |
| --- | --- | --- | --- |
| **TP53 SNVs** | 1.67 | 1.20-2.33 | **0.002** |

**Supplementary Table 5.** Multivariate analysis of pathway alterations in the cohorts (LDep vs. HDep).

Abbreviations: LDep, low deprivation neighborhoods; HDep, high deprivation neighborhoods; SNVs, Single Nucleotide Variants; CNVs, Copy Number Alterations; OR, Odds Ratio; C.I, Confidence Interval

|  | **HR** | **95% C.I.** | **P>\|z\|** |
| --- | --- | --- | --- |
| **Visceral** | 1.53 | 1.22-1.91 | **<0.001** |
|  |  |  |  |
| **Bone** | 1.35 | 1.05-1.74 | **0.020** |
|  |  |  |  |
| **Node** | 0.98 | 0.79-1.22 | 0.843 |
|  |  |  |  |
| **Soft tissue** | 1.62 | 1.26-2.08 | **<0.001** |
|  |  |  |  |
| **De novo** | 1.33 | 1.05-1.69 | **0.020** |
|  |  |  |  |
| **Therapy line** |  |  |  |
| 2 | 2.33 | 1.66-3.26 | **<0.001** |
| >=3 | 3.83 | 2.87-5.13 | **<0.001** |
|  |  |  |  |
| **Black** | 0.95 | 0.62-1.45 | 0.818 |
|  |  |  |  |
| **Age (≥60 vs <60)** | 1.03 | 0.83 – 1.28 | 0.809 |
|  |  |  |  |
| **Subtype (HR+/HER2- as reference)** |  |  |  |
| **HER2+** | 0.73 | 0.49 – 1.09 | 0.128 |
| **TNBC** | 1.89 | 1.44 – 2.48 | **<0.001** |

**Supplementary Table 6.** Univariate Cox proportional hazard analysis of clinical characteristics associated with overall survival in the LDep population.

Abbreviations: LDep, low deprivation neighborhoods; HDep, high deprivation neighborhoods; HR, Hazard Ratio; C.I, Confidence Interval; ET, Endocrine Therapy.

|  | **HR** | **95% C.I.** | **P>\|z\|** |
| --- | --- | --- | --- |
| **PIK3CA SNVs** | 1.25 | 1.00-1.57 | 0.049 |
|  |  |  |  |
| **ESR1 SNVs** | 2.05 | 1.61 – 2.60 | <0.001 |
|  |  |  |  |
| **TP53 SNVs** | 2.05 | 1.65-2.54 | <0.001 |
|  |  |  |  |
| **ARID1A SNVs** | 0.98 | 0.58-1.64 | 0.931 |
|  |  |  |  |
| **ERBB2 SNVs** | 1.34 | 0.79-2.29 | 0.282 |
|  |  |  |  |
| **PTEN SNVs** | 1.73 | 1.04-2.86 | 0.034 |
|  |  |  |  |
| **AKT1 SNVs** | 1.22 | 0.76-1.96 | 0.420 |
|  |  |  |  |
| **GATA3 SNVs** | 1.23 | 0.81-1.86 | 0.338 |
|  |  |  |  |
| **BRCA2 SNVs** | 0.85 | 0.45-1.59 | 0.602 |
|  |  |  |  |
| **KRAS SNVs** | 0.80 | 0.44-1.46 | 0.473 |
|  |  |  |  |
| **ATM SNVs** | 0.82 | 0.44-1.54 | 0.538 |
|  |  |  |  |
| **BRAF CNVs** | 2.59 | 1.66-4.03 | <0.001 |
|  |  |  |  |
| **FGFR1 CNVs** | 1.29 | 0.90-1.84 | 0.173 |
|  |  |  |  |
| **CCND1 CNVs** | 1.57 | 1.10-2.25 | 0.014 |
|  |  |  |  |
| **PIK3CA CNVs** | 2.14 | 1.54-2.97 | <0.001 |
|  |  |  |  |
| **EGFR CNVs** | 1.87 | 1.30-2.70 | 0.001 |
|  |  |  |  |
| **MYC CNVs** | 2.44 | 1.74-3.43 | <0.001 |
|  |  |  |  |
| **CCNE1 CNVs** | 2.08 | 1.32-3.28 | 0.002 |

**Supplementary Table 7.** Univariate Cox proportional hazards analysis of single gene alterations in the LDep population.

Abbreviations: LDep, low deprivation neighborhoods; HDep, high deprivation neighborhoods; HR, Hazard Ratio; SNVs, Single Nucleotide Variants; CNVs, Copy Number Alterations

|  | **HR** | **95% C.I.** | **P>\|z\|** |
| --- | --- | --- | --- |
| **Visceral** | 1.38 | 1.09-1.75 | 0.007 |
|  |  |  |  |
| **Bone** | 1.37 | 1.03 – 1.83 | 0.033 |
|  |  |  |  |
| **Soft tissue** | 1.64 | 1.24 – 2.15 | <0.001 |
|  |  |  |  |
| **Therapy line** |  |  |  |
| 2 | 2.28 | 1.60-3.24 | <0.001 |
| >=3 | 3.17 | 2.33-4.31 | <0.001 |
|  |  |  |  |
| **De novo** | 1.18 | 0.91-1.53 | 0.205 |
|  |  |  |  |
| **Subtype (HR+/HER2- as reference)** |  |  |  |
| HER2+ | 0.84 | 0.55 – 1.29 | 0.428 |
| TNBC | 2.28 | 1.65 – 3.16 | <0.001 |
|  |  |  |  |
| **ESR1SNVs** | 1.61 | 1.23 – 2.11 | <0.001 |
|  |  |  |  |
| **TP53 SNVs** | 1.67 | 1.33-2.11 | <0.001 |

**Supplementary Table 8.** Multivariate Cox proportional hazard regression analysis of clinical characteristics and SNVs associated with overall survival in the LDep population.

Abbreviations: LDep, low deprivation neighborhoods; HDep, high deprivation neighborhoods; SNVs, Single Nucleotide Variants; CNVs, HR, Hazard Ratio; C.I, Confidence Interval

|  | **HR** | **95% C.I.** | **P>\|z\|** |
| --- | --- | --- | --- |
| **Visceral** | 1.33 | 1.05-1.69 | 0.018 |
|  |  |  |  |
| **Bone** | 1.42 | 1.06 – 1.88 | 0.016 |
|  |  |  |  |
| **Soft tissue** | 1.64 | 1.25-2.14 | <0.001 |
|  |  |  |  |
| **Therapy line** |  |  |  |
| 2 | 2.31 | 1.62-3.28 | <0.001 |
| >=3 | 3.70 | 2.73-5.02 | <0.001 |
|  |  |  |  |
| **Subtype (HR+/HER2- as reference)** |  |  |  |
| HER2+ | 0.69 | 0.45 – 1.06 | 0.093 |
| TNBC | 2.14 | 1.57 – 2.92 | <0.001 |
|  |  |  |  |
| **De novo** | 1.19 | 0.92-1.95 | 0.181 |
|  |  |  |  |
| **BRAF CNVs** | 1.22 | 0.71-2.09 | 0.469 |
|  |  |  |  |
| **CCND1 CNVs** | 1.30 | 0.88-1.90 | 0.185 |
|  |  |  |  |
| **PIK3CA CNVs** | 1.74 | 1.19-2.55 | 0.005 |
|  |  |  |  |
| **EGFR CNVs** | 1.24 | 0.79-1.94 | 0.358 |
|  |  |  |  |
| **MYC CNVs** | 1.91 | 1.20-2.66 | 0.002 |
|  |  |  |  |
| **CCNE1 CNVs** | 1.10 | 0.79-2.25 | 0.283 |

**Supplementary Table 9.** Multivariate Cox proportional hazards regression analysis of clinical characteristics and CNVs associated with overall survival in the LDep population.

Abbreviations: LDep, low deprivation neighborhoods; HDep, high deprivation neighborhoods; CNVs, Copy Number Alterations; HR, Hazard Ratio; C.I, Confidence Interval

|  | **HR** | **95% C.I.** | **P>\|z\|** |
| --- | --- | --- | --- |
| **PI3K SNVs** | 1.31 | 1.06-1.63 | 0.014 |
|  |  |  |  |
| **ER SNVs** | 1.72 | 1.37-2.17 | <0.001 |
|  |  |  |  |
| **P53 SNVs** | 1.94 | 1.57-2.41 | <0.001 |
|  |  |  |  |
| **RTK SNVs** | 1.26 | 0.95-1.67 | 0.110 |
|  |  |  |  |
| **Cell Cycle SNVs** | 1.86 | 1.17-2.95 | 0.009 |
|  |  |  |  |
| **RTK/RAS CNVs** | 1.59 | 1.25-2.04 | <0.001 |
|  |  |  |  |
| **Cell Cycle CNVs** | 1.77 | 1.35-2.33 | <0.001 |
|  |  |  |  |
| **PI3K CNVs** | 2.14 | 1.54-2.97 | <0.001 |
|  |  |  |  |
| **MYC CNVs** | 2.44 | 1.74-3.43 | <0.001 |

**Supplementary Table 10.** Univariate Cox proportional hazards regression analysis of pathway alterations associated with overall survival in the LDep population.

Abbreviations: LDep, low deprivation neighborhoods; HDep, high deprivation neighborhoods; SNVs, Single Nucleotide Variants; CNVs, Copy Number Alterations; HR, Hazard Ratio; C.I, Confidence Interval

|  | **HR** | **95% C.I.** | **P>\|z\|** |
| --- | --- | --- | --- |
| **Visceral** | 1.39 | 1.10-1.76 | 0.007 |
|  |  |  |  |
| **Bone** | 1.36 | 1.01 – 1.82 | 0.042 |
|  |  |  |  |
| **Soft tissue** | 1.60 | 1.22-2.10 | <0.001 |
|  |  |  |  |
| **Therapy line** |  |  |  |
| 2 | 2.36 | 1.66-3.35 | <0.001 |
| >=3 | 3.29 | 2.43-4-47 | <0.001 |
|  |  |  |  |
| **De novo** | 1.17 | 0.91-1.52 | 0.226 |
|  |  |  |  |
| **Subtype (HR+/HER2- as reference)** |  |  |  |
| HER2+ | 0.85 | 0.55 – 1.32 | 0.476 |
| TNBC | 2.29 | 1.65 – 3.16 | <0.001 |
|  |  |  |  |
| **PI3K SNVs** | 1.17 | 0.93-1.49 | 0.180 |
|  |  |  |  |
| **ER SNVs** | 1.35 | 1.03-1.78 | 0.032 |
|  |  |  |  |
| **P53 SNVs** | 1.60 | 1.43-2.27 | <0.001 |
|  |  |  |  |
| **Cell Cycle SNVs** | 1.23 | 0.73-1.76 | 0.432 |

**Supplementary Table 11.** Multivariate Cox proportional hazards regression analysis of clinical characteristics and pathway SNVs associated with overall survival in the LDep population. Abbreviations: LDep, low deprivation neighborhoods; HDep, high deprivation neighborhoods; SNVs, Single Nucleotide Variants; HR, Hazard Ratio; C.I, Confidence Interval

|  | **HR** | **95% C.I.** | **P>\|z\|** |
| --- | --- | --- | --- |
| **Visceral** | 1.34 | 1.05-1.70 | 0.017 |
|  |  |  |  |
| **Bone** | 1.44 | 1.09-1.91 | 0.011 |
|  |  |  |  |
| **Soft tissue** | 1.60 | 1.23-2.09 | 0.001 |
|  |  |  |  |
| **Therapy line** |  |  |  |
| 2 | 2.29 | 1.62-3.27 | <0.001 |
| >=3 | 3.67 | 2.71-4.98 | <0.001 |
|  |  |  |  |
| **De novo** | 1.18 | 0.92-1.53 | 0.195 |
|  |  |  |  |
| **Subtype (HR+/HER2- as reference)** |  |  |  |
| HER2+ | 0.69 | 0.45 – 1.05 | 0.084 |
| TNBC | 2.14 | 1.58-2.90 | <0.001 |
|  |  |  |  |
| **RTK/RAS CNVs** | 1.13 | 0.82-1.55 | 0.459 |
|  |  |  |  |
| **Cell Cycle CNVs** | 1.21 | 0.87-1.67 | 0.258 |
|  |  |  |  |
| **PI3K CNVs** | 1.84 | 1.29-2.63 | <0.001 |
|  |  |  |  |
| **MYC CNVs** | 1.92 | 1.28 – 2.87 | 0.002 |

**Supplementary Table 12.** Multivariate Cox proportional hazards regression analysis of clinical characteristics and pathway CNVs associated with overall survival in the LDep population. Abbreviations: LDep, low deprivation neighborhoods; HDep, high deprivation neighborhoods; CNVs, Copy Number Alterations; HR, Hazard Ratio; C.I, Confidence Interval

|  | **HR** | **95% C.I.** | **P>\|z\|** |
| --- | --- | --- | --- |
| **Visceral** | 1.46 | 1.06-2.00 | 0.021 |
|  |  |  |  |
| **Bone** | 1.01 | 0.70-1.46 | 0.948 |
|  |  |  |  |
| **Node** | 1.26 | 0.90-1.78 | 0.182 |
|  |  |  |  |
| **Soft tissue** | 1.02 | 0.45-2.31 | 0.967 |
|  |  |  |  |
| **De novo** | 0.96 | 0.66-1.41 | 0.847 |
|  |  |  |  |
| **Therapy line** |  |  |  |
| 2 | 1.24 | 0.76-2.03 | 0.389 |
| >=3 | 1.79 | 1.22-2.62 | 0.003 |
|  |  |  |  |
| **Black** | 1.47 | 1.03-2.10 | 0.034 |
|  |  |  |  |
| **Age (≥60 vs <60)** | 1.06 | 0.76 – 1.46 | 0.736 |
|  |  |  |  |
| **Subtype (HR+/HER2- as reference)** |  |  |  |
| **HER2+** | 0.68 | 0.35 – 1.35 | 0.272 |
| **TNBC** | 2.04 | 1.33 – 3.14 | 0.001 |

**Supplementary Table 13.** Univariate Cox proportional hazards regression analysis of clinical characteristics associated with overall survival in the HDep population.

Abbreviations: LDep, low deprivation neighborhoods; HDep, high deprivation neighborhoods; HR, Hazard Ratio; C.I, Confidence Interval; ET, Endocrine Therapy.

|  | **HR** | **95% C.I.** | **P>\|z\|** |
| --- | --- | --- | --- |
| **PIK3CA SNVs** | 1.65 | 1.20-2.28 | 0.002 |
|  |  |  |  |
| **ESR1 SNVs** | 1.49 | 1.04-2.15 | 0.031 |
|  |  |  |  |
| **TP53 SNVs** | 1.79 | 1.30-2.46 | <0.001 |
|  |  |  |  |
| **ARID1A SNVs** | 0.76 | 0.39-1.50 | 0.429 |
|  |  |  |  |
| **ERBB2 SNVs** | 2.11 | 1.03-4.32 | 0.041 |
|  |  |  |  |
| **PTEN SNVs** | 2.13 | 1.04-4.35 | 0.038 |
|  |  |  |  |
| **AKT1 SNVs** | 6.27 | 2.27-17.33 | <0.001 |
|  |  |  |  |
| **GATA3 SNVs** | 1.06 | 0.52-2.16 | 0.879 |
|  |  |  |  |
| **BRCA2 SNVs** | 0.71 | 0.26-1.93 | 0.505 |
|  |  |  |  |
| **KRAS SNVs** | 1.09 | 0.40-2.96 | 0.869 |
|  |  |  |  |
| **ATM SNVs** | 1.06 | 0.43-2.58 | 0.904 |
|  |  |  |  |
| **BRAF CNVs** | 5.36 | 2.91-9.87 | <0.001 |
|  |  |  |  |
| **FGFR1 CNVs** | 2.34 | 1.40-3.91 | 0.001 |
|  |  |  |  |
| **CCND1 CNVs** | 2.81 | 1.61-4.90 | <0.001 |
|  |  |  |  |
| **PIK3CA CNVs** | 3.23 | 1.84-5.66 | <0.001 |
|  |  |  |  |
| **EGFR CNVs** | 2.33 | 1.44-3.79 | 0.001 |
|  |  |  |  |
| **MYC CNVs** | 4.53 | 2.64-7.76 | <0.001 |
|  |  |  |  |
| **CCNE1 CNVs** | 1.90 | 1.05-3.45 | 0.002 |

**Supplementary Table 14.** Univariate Cox regression analysis of SNVs in the HDep population. Abbreviations: LDep, low deprivation neighborhoods; HDep, high deprivation neighborhoods; HR, Hazard Ratio; SNVs, Single Nucleotide Variants; C.I, Confidence Interval

|  | **HR** | **95% C.I.** | **P>\|z\|** |
| --- | --- | --- | --- |
| **Visceral** | 1.47 | 1.01 – 2.13 | 0.042 |
|  |  |  |  |
| **Therapy line** |  |  |  |
| 2 | 0.98 | 0.58 – 1.67 | 0.947 |
| >=3 | 1.47 | 0.94 - 2.32 | 0.090 |
|  |  |  |  |
| **Black** | 1.37 | 0.91 – 2.05 | 0.127 |
|  |  |  |  |
| **Subtype (HR+/HER2- as reference)** |  |  |  |
| **HER2+** | 0.84 | 0.42 – 1.69 | 0.624 |
| **TNBC** | 3.17 | 1.86 – 5.43 | <0.001 |
|  |  |  |  |
| **PIK3CA SNVs** | 1.59 | 1.09 – 2.33 | 0.016 |
|  |  |  |  |
| **ESR1 SNVs** | 1.25 | 0.82 – 1.92 | 0.305 |
|  |  |  |  |
| **TP53 SNVs** | 1.38 | 0.97 – 1.98 | 0.077 |
|  |  |  |  |
| **ERBB2 SNVs** | 1.43 | 0.60 – 3.43 | 0.417 |
|  |  |  |  |
| **PTEN SNVs** | 1.99 | 0.93-4.28 | 0.077 |
|  |  |  |  |
| **AKT1 SNVs** | 7.88 | 2.68 – 23.15 | <0.001 |

**Supplementary Table 15.** Multivariate Cox proportional hazards regression analysis of clinical characteristics and SNVs associated with overall survival in the HDep population. Abbreviations: LDep, low deprivation neighborhoods; HDep, high deprivation neighborhoods; SNVs, Single Nucleotide Variants; HR, Hazard Ratio; C.I, Confidence Interval

|  | **HR** | **95% C.I.** | **P>\|z\|** |
| --- | --- | --- | --- |
| **Visceral** | 1.24 | 0.86-1.78 | 0.254 |
|  |  |  |  |
| **Therapy line** |  |  |  |
| 2 | 1.09 | 0.66 – 1.81 | 0.732 |
| >=3 | 1.63 | 1.06 – 2.50 | 0.026 |
|  |  |  |  |
| **Black** | 1.27 | 0.87 – 1.87 | 0.219 |
|  |  |  |  |
| **Subtype (HR+/HER2- as reference)** |  |  |  |
| **HER2+** | 0.66 | 0.33 – 1.35 | 0.258 |
| **TNBC** | 2.68 | 1.53 – 4.71 | 0.001 |
|  |  |  |  |
| **BRAF CNVs** | 1.94 | 0.78 – 4.80 | 0.152 |
|  |  |  |  |
| **FGFR1 CNVs** | 1.98 | 1.05 – 3.76 | 0.036 |
|  |  |  |  |
| **CCND1 CNVs** | 1.62 | 0.83 – 3.14 | 0.154 |
|  |  |  |  |
| **PIK3CA CNVs** | 1.45 | 0.66 – 3.18 | 0.357 |
|  |  |  |  |
| **EGFR CNVs** | 0.83 | 0.43 – 1.62 | 0.581 |
|  |  |  |  |
| **MYC CNVs** | 3.21 | 1.57-6.60 | 0.001 |

**Supplementary Table 16.** Multivariate Cox proportional hazards regression analysis of clinical characteristics and CNVs associated with overall survival in the HDep population. Abbreviations: LDep, low deprivation neighborhoods; HDep, high deprivation neighborhoods;, Copy Number Alterations; HR, Hazard Ratio; C.I, Confidence Interval

|  | **HR** | **95% C.I.** | **P>\|z\|** |
| --- | --- | --- | --- |
| **PI3K SNVs** | 1.84 | 1.33-2.53 | <0.001 |
|  |  |  |  |
| **ER SNVs** | 1.49 | 1.05-2.12 | 0.024 |
|  |  |  |  |
| **P53 SNVs** | 1.73 | 1.26-2.38 | 0.001 |
|  |  |  |  |
| **RTK/RAS SNVs** | 1.75 | 1.13-2.70 | 0.013 |
|  |  |  |  |
| **Cell Cycle SNVs** | 1.95 | 0.95-3.97 | 0.068 |
|  |  |  |  |
| **RTK/RAS CNVs** | 2.59 | 1.82-3.67 | <0.001 |
|  |  |  |  |
| **Cell Cycle CNVs** | 2.42 | 1.60-3.67 | <0.001 |
|  |  |  |  |
| **PI3K CNVs** | 3.23 | 1.84-5.66 | <0.001 |
|  |  |  |  |
| **MYC CNVs** | 4.53 | 2.64-7.76 | <0.001 |

**Supplementary Table 17.** Univariate Cox proportional hazards regression analysis of pathway alterations associated with overall survival in the HDep population. Abbreviations: LDep, low deprivation neighborhoods; HDep, high deprivation neighborhoods; HR, Hazard Ratio; SNVs, Single Nucleotide Variants; CNVs, Copy Number Alterations; C.I, Confidence Interval

|  | **HR** | **95% C.I.** | **P>\|z\|** |
| --- | --- | --- | --- |
| **Visceral** | 1.50 | 1.04 – 2.16 | 0.030 |
|  |  |  |  |
| **Therapy line** |  |  |  |
| 2 | 0.87 | 0.51 – 1.47 | 0.607 |
| >=3 | 1.39 | 0.90 – 2.15 | 0.129 |
|  |  |  |  |
| **Black** | 1.34 | 0.89 – 2.03 | 0.157 |
|  |  |  |  |
| **PI3K SNVs** | 1.88 | 1.32 – 2.70 | 0.001 |
|  |  |  |  |
| **ER SNVs** | 1.41 | 0.94 – 2.13 | 0.100 |
|  |  |  |  |
| **P53 SNVs** | 1.30 | 0.91 – 1.86 | 0.146 |
|  |  |  |  |
| **RTK/RAS SNVs** | 1.61 | 0.98 – 2.64 | 0.060 |

**Supplementary Table 18.** Multivariate Cox proportional hazards regression analysis of clinical characteristics and pathways SNVs associated with overall survival in the HDep population. Abbreviations: LDep, low deprivation neighborhoods; HDep, high deprivation neighborhoods; HR, Hazard Ratio; SNVs, Single Nucleotide Variants; CNVs, Copy Number Alterations; C.I, Confidence Interval

|  | **HR** | **95% C.I.** | **P>\|z\|** |
| --- | --- | --- | --- |
| **Visceral** | 1.31 | 0.91 – 1.87 | 0.141 |
|  |  |  |  |
| **Therapy line** |  |  |  |
| 2 | 1.09 | 0.65 – 1.82 | 0.736 |
| >=3 | 1.52 | 0.99 – 2.31 | 0.051 |
|  |  |  |  |
| **Black** | 1.27 | 0.86-1.82 | 0.227 |
|  |  |  |  |
| **RTK/RAS CNVs** | 1.66 | 1.05 – 2.62 | 0.028 |
|  |  |  |  |
| **Cell Cycle CNVs** | 0.99 | 0.55 – 1.79 | 0.994 |
|  |  |  |  |
| **PI3K CNVs** | 1.30 | 0.63 – 2.71 | 0.481 |
|  |  |  |  |
| **MYC CNVs** | 2.92 | 1.37 – 6.25 | 0.006 |

**Supplementary Table 19.** Multivariate Cox proportional hazards regression analysis of clinical characteristics and pathways CNVs associated with overall survival in the HDep population. Abbreviations: LDep, low deprivation neighborhoods; HDep, high deprivation neighborhoods; SNVs, Single Nucleotide Variants; CNVs, Copy Number Alterations; HR, Hazard Ratio; C.I, Confidence Interval
